# EXPLORING THE EFFICACY AND EFFECTIVENESS OF REMINISCENCE USING THREE-DIMENSIONAL PRINTED OBJECTS

**DOI:** 10.1093/geroni/igz038.1129

**Published:** 2019-11-08

**Authors:** Keith A Anderson, Marla Berg-Weger, Tom Plocher, Anne Farina, Joseph E Gaugler

**Affiliations:** 1 University of Montana, Missoula, Montana, United States; 2 Saint Louis University, St. Louis, Missouri, United States; 3 Moai Technologies, Maple Grove, Minnesota, United States; 4 University of Minnesota - School of Public Health, Division of Health Policy and Management, Minneapolis, Minnesota, United States

## Abstract

Reminiscence therapy (RT) is a form of person-centered care that involves the exploration of past activities, events, and experiences with another person or in a small group. Research on RT has found an array of benefits for older adults, including improvements in mood, quality of life, social interaction, cognition, and memory. In this presentation, the researchers report on Phases I and II of an evaluation of a reminiscence using three-dimensional (3D) printed objects from older adults’ pasts. Advances in 3D printing technology now allow researchers to create scale replicas of cherished items from peoples’ pasts, such as toys, bicycles, pets, automobiles, boats, and houses. In Phase I, the researchers evaluated the efficacy of incorporating 3D objects in reminiscence using a parallel convergent mixed methods design. Participants agreed or strongly agreed that the 3D object reminiscence was well-received (88.9%), facilitated reminiscence (83.3%), increased engagement and alertness (72.3%). Qualitative data identified additional benefits of the use of 3D objects in reminiscence, including increases in social engagement and interactions with staff and family members. In Phase II, the researchers are evaluating the effectiveness of a formal 3D reminiscence intervention using a randomized control trial of 175 older adults. The researchers hypothesize that 3D object reminiscence will be more effective than reminiscence using verbal cues in stimulating memory and enhancing cognition, engagement, mood, and quality of life. Preliminary findings from Phase II will be reported along with the findings from Phase I.

